# Long non-coding RNA DBCCR1-003 regulate the expression of DBCCR1 via DNMT1 in bladder cancer

**DOI:** 10.1186/s12935-016-0356-8

**Published:** 2016-10-18

**Authors:** Defeng Qi, Jinhui Li, Biao Que, Jialin Su, Mengxi Li, Chaofeng Zhang, Mei Yang, Guoren Zhou, Weidong Ji

**Affiliations:** 1 Guangdong Key Laboratory of Urology, Department of Urology, Minimally Invasive Surgery Center, The First Affiliated Hospital of Guangzhou Medical University, Kangda Road 1#, Haizhu District, Guangzhou, 510230 Guangdong China; 2 The First Affiliated Hospital, Center for Translational Medicine, Sun Yat-sen University, Guangzhou, 510080 China; 3 The Affiliated Hospital of School of Medicine of Ningbo University, Zhejiang, 315000 China; 4 Breast Disease Center, Guangdong Women and Children Hospital of Guangzhou Medical University, Guangzhou, 510010 Guangdong China; 5 Department of General Surgery, General Hospital of Guangzhou Military Command of PLA, Guangzhou, 510010 Guangdong China; 6 Department of Medical Oncology, Jiangsu Cancer Hospital, Nanjing, 210009 Jiangsu China

**Keywords:** lncRNA, DBCCR1-003, DBCCR1, DNMT1, Bladder cancer

## Abstract

**Background:**

Many long non coding RNAs have been identified as key modulators in cancer development. A lncRNA, DBCCR1-003, derived from the locus of tumor suppressor gene DBCCR1 (deleted in bladder cancer chromosome region 1), has unknown function. In the present study, we explored function and molecular mechanism of DBCCR1-003 in bladder cancer (BC) development.

**Methods:**

We evaluated the expression levels of DBCCR1-003 in tissues and cells with western blot and quantitative real-time polymerase chain reaction. Multiple approaches including chromatin immunoprecipitation assay and RNA immunoprecipitation were used to confirm the direct binding of DBCCR1-003 to DNMT1. The recombinant vector overexpressing DBCCR1-003 was constructed. Cell proliferation assay, colony formation assay and flow cytometric analysis were employed to measure the role of DBCCR1-003 in regulation of cell proliferation, cycle and apoptosis.

**Results:**

Firstly we detected the expression of DBCCR1-003, DBCCR1, DNMT1 (DNA methyltransferase 1) and DNA methylation in the promoter of DBCCR1. We found low expression of DBCCR1-003, same as DBCCR1, while high expression of DNMT1 and hypermethylation of DBCCR1 gene promoter in BC tissues and T24 cells line. Further studies revealed that treatment of DNMT inhibitor, 5-aza-2-deoxycytidine(DAC), or overexpression of DBCCR1-003 led to increased DBCCR1 expression by reversion of promoter hypermethylation and DNMT1 binding to DBCCR1 promoter in T24 cells. Importantly, RNA immunoprecipitation (RIP) showed that DBCCR1-003 physically associates with DNMT1. The binding of them was increased with the inhibition of DBCCR1 promoter methylation, indicating that DBCCR1-003 may bind to DNMT1 and prevent DNMT1-mediated the methylation of DBCCR1. Furthermore, overexpression of DBCCR1-003 resulted in significant inhibition of T24 cells growth through the inducing G0/G1 arrest and apoptosis.

**Conclusions:**

Taken together, these findings demonstrated that a novel tumor suppressor DBCCR1-003 regulates the expression of DBCCR1 via binding to DNMT1 and preventing DNMT1-mediated the methylation of DBCCR1 in BC. LncRNA DBCCR1-003 may serve as a novel biomarker and therapeutic target for BC in future cancer clinic.

**Electronic supplementary material:**

The online version of this article (doi:10.1186/s12935-016-0356-8) contains supplementary material, which is available to authorized users.

## Background

Bladder cancer is the most common cancer of urinary system in china. Although approximately 70 % of patients will preliminary diagnosis as nonmuscle-invasive BC (NMIBC), 50–70 % of patients will recur and about 10–20 % will progressed to muscle-invasive BC (MIBC) [1]. So, exploring the early diagnostic and prognostic markers for bladder cancer and molecular mechanisms involving in bladder cancer is significant for raising the survival rates of bladder cancer patients. Nowadays, many mechanisms involving in bladder cancer have been confirm by studies, such as the activation of proto-oncogene, the inactivation of tumor suppressor gene (point mutation, rearrangement and deficiency), chromosome abnormality and so on. Since many mechanisms are still unclear, there is a need to further understand the molecular mechanisms involving in bladder cancer development for exploring the effective therapeutic modalities and early detection approach.

The long non-coding RNA (lncRNA) is a kind of RNA with size over 200 nt and has no protein-coding capacity [2]. Unlike classical coding genes, which function by translated into protein molecules, lncRNAs play a key roles in regulation of various biological process in the shape of RNA and have exhibited less evolutionary constraint [3, 4]. The expression of genes has been revealed to be regulated by lncRNAs in kinds of different approaches including repression of neighboring (*cis*) genes, distant (*trans*) via histone modification, and through interaction with miRNAs [5–7]. Increasing evidences have indicated that lncRNAs were closely involved in carcinogenesis and have the potential to be early tumor diagnostic markers and molecular-targeted therapy sites [5, 8, 9]. In BC, lncRNAs are associated with carcinogenesis, development and prognosis [10, 11]. Xue et al. found that the low expression of lncRNA MDC1-AS was involved in BC by up-regulation of its antisense tumor suppressing gene MDC1 [12]. He et al. revealed a new lncRNA, linc-UBC1 (Up-regulated in bladder cancer 1), was over-expressed in BC tissues and it was associated with lymph node metastasis and poor survival [13]. Accumulating evidence indicated that abnormal expression of lncRNAs had a close relationship with cancers [14, 15]. So, lncRNAs have the potential to be diagnostic markers and therapeutic targets for BC in the clinic.

It is generally accepted that lncRNAs, as any other protein-coding gene, undergo the same regulatory mechanisms including epigenetic regulation [16, 17]. As one of the most extensively studied epigenetic change, Aberrant DNA methylation is associated with various biological processes including cancer [18–21]. It is a procedure of chemical modification which will specifically methylate the cytosines located 5′ to guanosines in CpG dinucleotides and give rise to 5-methylcytosine (m5C) via the DNA methyltransferases (DNMTs) [22]. As one of the DNMTs, DNMT1 has the power to maintain the methylation of newly replicated DNA. Studies have demonstrated that lncRNAs could associate with DNMT1, contributing to the expression of gene and aberrant DNA methylation during the tumorigenesis [23]. However, whether the tumorigenesis and development of BC can affected by lncRNAs via DNMT1 or not and the molecular mechanism involved in the process are unclear.

Lately, studies had identified that lncRNA may directly associate with DNMT1 through binding to it, and prevent the methylation of tumor suppressor gene [24]. In colon cancer, lncRNA with low expression was also found to regulate epigenetic modifications and the expression of specific gene by assembles DNMT1 at specific genomic sites [25]. To examine if this function be suitable for BC, we investigated a well-studied tumor suppressor gene DBCCR1 (deleted in bladder cancer chromosome region 1) with a methylation sensitive and lncRNA DBCCR1-003 (name got from the database of lncRNAs, transcript ID:ENST00000482797) arising from the locus of DBCCR1. DBCCR1 is located at chromosome 9q32-33 identified by loss of heterozygosity (LOH) studies of human BC to act as a tumor suppressor gene [26]. Performing demethylation experiments in BC cells resulted in the re-expression of DBCCR1 mRNA indicating that DBCCR1 expression is silenced by hypermethylation [27]. These features make DBCCR1 be a good candidate for our study.

To prove our hypothesis, we first test the expression of DBCCR1-003, DBCCR1 and DNMT1 as well as methylation state of DBCCR1 promoter in BC cells and tissues. Then, we investigated the expression change of DBCCR1-003, DBCCR1 and DNMT1 and methylation dynamics of DBCCR1 by knock-in DBCCR1-003 and conducting demethylation treatment in BC cells. The function of DBCCR1-003 was determined by using cell proliferation, clone formation assay, cell apoptosis and cell cycle analysis. RNA immunoprecipitation (RIP) was conducted to confirm if DBCCR1-003 physically associates with DNMT1. Chromatin immunoprecipitation (ChIP) was performed to measure the binding of DNMT1 in DBCCR1 CpG island promoter. According to the research conclusions above and our previous results, this study was designed to detect that whether the tumorigenesis and development of BC can affected by lncRNA DBCCR1-003 via DNMT1 or not, and investigate the possible underlying molecular mechanism involved in the process.

## Methods

### Patients and tissue samples

This study has been reviewed by the Institutional Review Board (IRB) of Guangzhou Medical University with the approval number of GMU-IRB#: 2015-11. After being acquainted with the aim and the methods used in the study, each of the patients included in the study signed a written informed consent form. Between January 2012 and May 2015, a total of 24 specimens were obtained from patients undergoing BC surgery at the first affiliated Hospital of Guangzhou medical University (Kangda Road 1#, Haizhu District, Guangzhou, Guangdong, China). None received any antitumoral treatment prior to tumour sampling. All specimens were pathologically graded and staged according to the TNM and World Health Organization classification. The histopathological classification of urinary BC was confirmed by two independent histopathologists. A total of 24 adjacent tissues of cancer from matched patients were collected as control group. The patients included 14 males and 10 females. The median patient age was 69 years with range 47 to 90 years. More details of characteristics are classified into Table 1.

**Table 1:** Clinicopathologic features of BC patients and the levels of DBCCR1-003 and DNMT1 expression in the cancer tissues

Characteristic	Number of patients	Molecular expression level			
		DBCCR1-003^c^	p value	DNMT1^d^	p value
All patients	24 (100 %)				
Gender^a^			0.403		0.639
Females	10 (41.6 %)	4.61 (1.10–4.19)		1.68 (1.2–2.39)	
Males	14 (58.4 %)	1.60 (0.56–3.70)		1.87 (0.65–3.43)	
Age at diagnosis^a^			0.887		0.931
≤70 years	12 (50.0 %)	2.0 (0.58–3.70)		1.85 (0.65–3.43)	
>70 years	12 (50.0 %)	1.91 (0.56–4.19)		1.74 (0.97–2.39)	
Pathologic grade^a^			0.02*		0.035*
Low grade	8 (33.3 %)	2.90 (1.39–4.19)		1.4 (0.97–2.19)	
High grade	16 (66.7 %)	1.39 (0.57–2.81)		1.98 (1.15–3.43)	
Pathologic stage^b^			0.14		0.037*
pTa	5 (20.8 %)	3.39 (2.51–4.19)		1.26 (0.65–1.84)	
pT1	13 (54.2 %)	1.84 (0.66–3.70)		1.74 (1.0–2.39)	
≥pT2	6 (25.0 %)	1.02 (0.57–1.57)		2.34 (1.35–2.71)	

* Significant difference

^a^Evaluated with the Mann–Whitney U-test

^b^Evaluated with the Kruskal–Wallis test

^c^Levels of DBCCR1-003 expression were determined by qRT–PCR assay, and normalized to the U6 levels using the 2^−△CT△CT^ method

^d^Levels of DNMT1 expression were determined by qRT–PCR assay, and normalized to the GAPDH levels using the 2^−△CT△CT^ method

### Cell culture and transfection

Both of the human urinary bladder transitional carcinoma cell lines T24 and human bladder epithelial immortalized cell lines SV-HUC-1 were purchased from American type culture collection (ATCC). The T24 cells was cultured in RPMI 1640, and SV-HUC-1 cells was cultured in F12K. All medium were supplemented with 10 % fetal bovine serum (Gibco, USA), in a humidified air atmosphere of 5 % CO2 at 37 °C. We had used 0.25 % trypsin (with 1 mM EDTA) (Invitrogen, Carlsbad, CA) to harvest the cells for further experiment. Cells were grown in polystyrene 25 cm^2^ dishes and transfected with 3.0 μg of DNA using 30 μl of Lipofectamine transfection reagent (Life Technologies) according to the manufacturer’s recommendations for 6 h.

### Construction of vectors

Plasmid cDNA-DBCCR1-003 was constructed by introducing *Spe*I-*Not*I fragment containing the DBCCR1-003 cDNA into the same site in LentiORF PLEX-MCS vector. The recombinant vectors were designated as LentiORF PLEX-MCS-DBCCR1-003 and identified by sequencing. At the same time, we also constructed the control vector named PLEX-MCS-control. Both of the vectors were transfected in 293FT cells, respectively. Generated virus particles subsequently infected T24 cells, the positive clones were obtained following puromycin selection. The stable cell lines achieved were correspondingly designated as Lenti-DBCCR1-003(L-D3) and control Lenti-vector (L-C).

### Real-time quantitative PCR

Total RNA was extracted using the Trizol Reagent (Invitrogen, USA) according to the instructions. The RNA purity and concentration were determined by the UV spectrophotometer. cDNA was reversibly transcribed from the extracted total RNA using an MMLV reagent kit (TaKaRa, Japan) and the primers were designed as Additional file 1: Table S1. The expression of the filtered lncRNAs and their associated encoding genes was measured using SYBR real-time PCR (qPCR) (Takara Bio, Otsu, Japan) according to the manufacturer’s instructions. PCR was then carried out as follows: denaturing at 95 °C for 20 s, 40 cycles of 10 s at 95 °C, 20 s at 58 °C and 30 s at 72 °C.

### 5-Aza-2′-deoxycytidine (DAC) treatment

T24 cells were seeded at 50 % confluence 6 h before treatment. The doses of DAC (Sigma, St Louis, MO) for T24 cells were 12.5 μmol/l. The cell was treated with the designated doses for 48 h, and the confluence of the collected cell was never greater than 80 %.

### Western blot analysis

Cultured cells were collected and washed three with 1 ml of PBS. After cracked with protein lysis buffer (50 mM Tris (pH 7.4), 150 mM NaCl, 1 %Triton X-100, 1 % sodium deoxycholate, 0.1 % SDS, sodium orthovanadate, sodium fluoride, EDTA, leupeptin), cells were collected in a centrifuge tube. Cell lysates were centrifuged at 13,000×*g* for 15 min at 4 °C and insoluble debris was discarded. Soluble proteins were subjected to 8 % SDS-PAGE, after electrophoresis, the proteins were transferred onto PVDF membrane and detected by immunolabeling with primary and secondary anti-bodies. In this experiment, we made GAPDH as the internal reference. Protein bands were quantified using Chemiluminescence with Koda film.

### Methylation-specific PCR (MSP)

Primer sequences for DBCCR1 were list in Additional file 1: Table S1. Genomic DNA of tissues were extracted from frozen specimens and digested by proteinase K followed by standard phenol/chloroform purification and ethanol precipitation. Reagents required for the bisulfite modification of DNA were supplied in the EZ DNA Methylation-Gold Kit (ZYMO RESEARCH). The process was performed according to the manufacturer’s recommendations. 1 μg of DNA was modified with sodium bisulfite to convert all unmethylated (but not methylated) cytosine to uracil followed by amplification with primers specific for methylated versus unmethylated DNA [28]. DNA from normal lymphocytes was used as control. Water was also used as negative control for contamination. Methylation status of each tumor was evaluated in triplicate for reproducible in MSP. PCR products were electrophoresed on a 2 % agarose gel for analysis.

### Cell proliferation analysis

Cells were plated into a new dish. 1 × 10^5^ cells were plated in triplicate and harvested at the indicated time points: 24, 48, 72, and 96 h. The number of cells was determined using an Auto T4 Plus Cell Counter (Nexcelom Bioscience, USA). Triplicate plates were counted for each cell lines.

### Colony formation assay

Cell survival was measured using a standard colony forming assay. Cells were seeded onto six-well plates at 400 cells per well. One week later, colonies were fixed with 100 % methanol for 15 min and stained with 0.1 % crystal violet for 20 min. Microscopic colonies composed of more than approximately 50 cells were counted as having grown from surviving cells.

### Apoptosis determination by flow cytometry

The cells were harvested by centrifugation for 3 min at 1000 rpm and were resuspended in binding buffer. Aliquots containing 1 × 10^5^ cells in 190 μl of buffer were stained with 10 μl of PI solution and with 5 μl of Annexin V-FITC (eBioscience, USA) for 10 min at room temperature. The excitation and emission wavelengths of FITC was FL1 PMT with 515–545 nm, and PI was FL3 with 650 nm. Then Flow cytometric analysis was performed using a flow cytometer (BD, USA) to detect the cell apoptosis.

### Cell cycle analysis

Cells were collected by trypsin method, washed with PBS, fixed overnight at 4 °C in 70 % ethanol. They were then washed in cold PBS and resuspended in 50 μg/ml propidium iodide and RNase A (50 μg/ml). The cell suspension was incubated in a 37 °C water bath for 1 h and cell cycle distribution was determined by flow cytometry. The cell cycle phase quantification was performed using ModFit LT to detect the cell apoptosis.

### Chromatin immunoprecipitation (ChIP) assay

Chromatin immunoprecipitation was performed with EZ-Magna ChIP A/G kit (Millipore) according to manufacturer’s instructions. Briefly, protein extract form 1 × 10^7^ cells were used for each reaction. Proteins were cross-linked to DNA by adding formaldehyde to the cell culture medium to a final concentration of 1 % at room temperature for 10 min and quenched by addition of 0.125 M glycine for 5 min at room temperature. The nucleus was isolated with nuclear lysis buffer (Millipore) supplemented with protease inhibitor cocktail (Millipore). Cells were sonicated and sheared to yield fragments between 200 and 1000 bp. 5 μg of either anti-DNMT1 (Abcam), Normal mouse IgG (the negative control) and anti-RNA polII (Millipore) (the positive control) was added to the sonicated samples and incubated at 4 °C overnight with rotation. Immune complexes were collected with Protein A/G agarose beads and washed with low salt buffer, high salt buffer, LiCl buffer and TE buffer to remove nonspecific binding. Protein/DNA complex was reverse cross-linked and DNA was purified using spin columns. Purified DNA was detected with quantitative PCR. Primers for ChIP-qPCR were listed in Additional file 1: Table S1.

### RNA immunoprecipitation (RIP) assay

RIP was conducted as described in [49]. Briefly, 1 × 10^7^ cells were harvested and lysed in complete RIP lysis buffer. Resuspended nuclear fraction was sheared by a homogenizer and sonicated. Antibodies against DNMT1 (Abcam) and IgG (Sigma-Aldrich) were incubated with magnetic beads (Protein A or G) for 1 h and the nuclear lysates were incubated overnight with rotation. Samples were incubated with Proteinase K and dealed with QIAamp MinElut Virus Spin kit (QIAGEN, GER) to isolated RNA. Purified RNA was reverse transcribed into cDNA by random primer (GeneCopoeia, USA), and detected with quantitative PCR. The binding of ecCEBPA and DNMT1 which had been identified by studies was used as a positive control [24], and normal mouse IgG was used as a negative control. Primers for RIP-qPCR were listed in Additional file 1: Table S1.

### Statistical analysis

All quantified data were analyzed by the SPSS 13 statistical software. Statistical significance was measured by Student’s *t* test and Mann–Whitney U-test. The relationship between the expression level of DBCCR1-003 and clinicopathologic parameters were analyzed using the Mann–Whitney U-test when comparing the differences between two groups, and using the Kruskal–Wallis test when comparing the differences among three or more groups. All p values <0.05 were considered significant.

## Results

### DBCCR1 and DBCCR1-003 are down-regulated in BC cells and tumor tissues

**Fig. 1:**
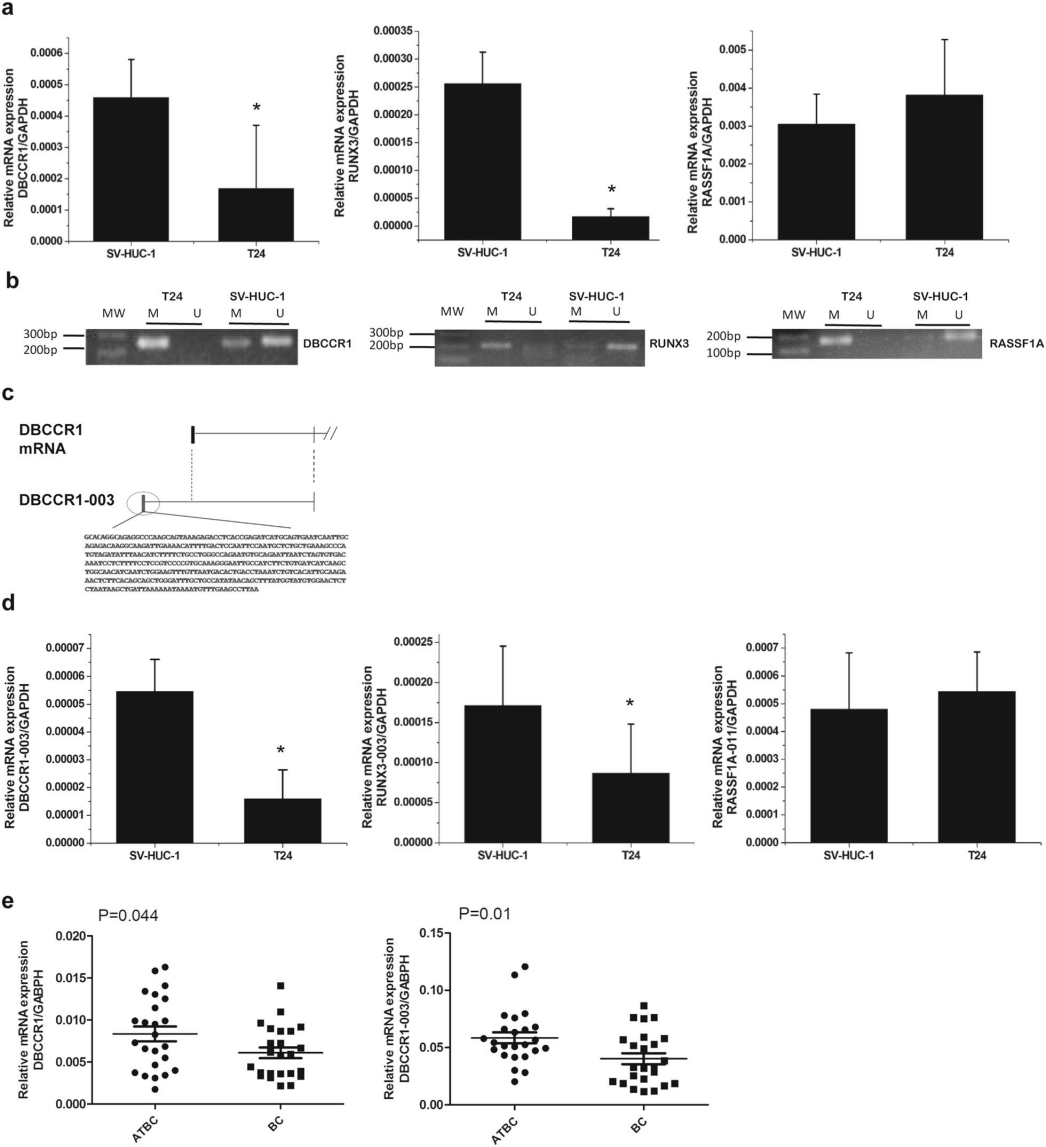
The selection of target lncRNA. **a** Relative expression of DBCCR1, RUNX3 and RASSF1A in T24 cells compared to SV-HUC-1 cells were measured by real-time PCR. **b** The methylation status of DBCCR1, RUNX3 and RASSF1A promoter in T24 cells and SV-HUC-1 cells were measured by MSP. *U* unmethylated status; *M* methylated status. **c** Schematic view of lncRNAs DBCCR1-003. The region in* black* indicates the exon and the region marking with *circular box* indicate the specific sequences of lncRNAs DBCCR1-003. **d** Relative expression of DBCCR1-003, RUNX3-003 and RASSF1A-011 in T24 cells compared to SV-HUC-1 cells were measured by real-time PCR. **e** Relative expression levels of DBCCR1 and DBCCR1-003 in 24 pairs of bladder cancer tumors (BC) and the matched adjacent tissues of bladder cancer (ATBT) were determined by real-time PCR. Results represent the mean + SD from three independent experiments.* Asterisk* indicate significant difference at p < 0.05

To determine a lncRNA regulates BC by mediating the expression of tumor suppressor gene via DNA methylation, we firstly chosen and tested three tumor suppressor genes: RASSF1A, RUNX3 and DBCCR1. All of them were down-regulated by the hypermethylation of promoter region in BC [26, 29, 30]. The expression level and methylation status of these genes in T24 cells and SV-HUC-1 cells were determined by SYBR real-time PCR and MSP, respectively. The results showed that DBCCR1 and RUNX3 were hypermethylation with a relative low expression in T24 cells compared to SV-HUC-1 cells, whereas RASSF1A was hypermethylation with a relative high expression (Fig. 1a, b). Then, by searching the database of Ensembl (http://asia.ensembl.org/index.html), we found non-coding RNAs from the transcripts of RASSF1A, RUNX3 and DBCCR1 and analyzed the homology between them and their sources via BLAST, finally selecting three non-coding RNAs with specific sequences: RASSF1A-011, RUNX3-003 (Additional file 1: Fig. S1) and DBCCR1-003 (Fig. 1c). Similar to their corresponding tumor suppressor genes, the expression level of RUNX3-003 and DBCCR1-003 were significantly decreased but RASSF1A-011 was increased in T24 cells when compared with SV-HUC-1 cells (Fig. 1d). What’s more, the expression difference in DBCCR1-003 was more obvious. We further measured the expression level of DBCCR1 and DBCCR1-003 in 24 pairs of BC tissue specimens and matched adjacent tissues of BC. Consistent with the results in cells, DBCCR1 and DBCCR1-003 had a relatively lower expression in BC tissues when compared with the matched adjacent tissues of BC (Fig. 1e). Based on the experimental results, we choose the DBCCR1-003 as target lncRNA for further studies. To analysis the Clinicopathologic features of BC patients, we found the lower DBCCR1-003 expression levels was significantly correlated with the BC grade, but not patient gender, stage and age (Table 1).

### DBCCR1-003 regulates the expression of DBCCR1 via DNA hypermethylation

**Fig. 2:**
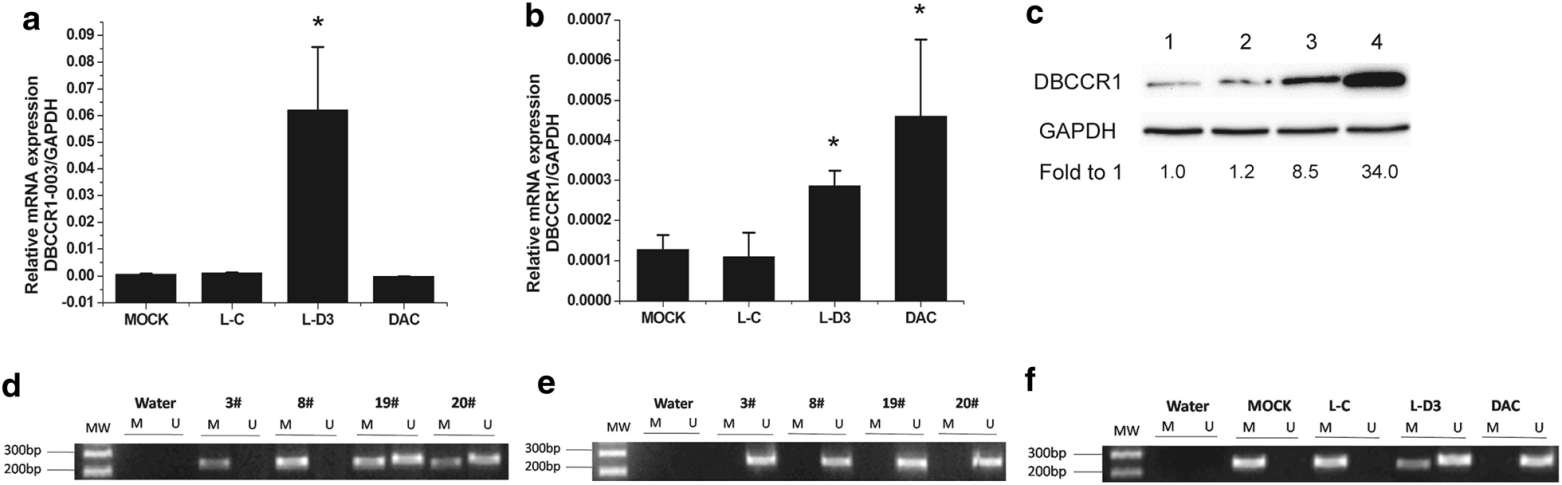
DBCCR1-003 regulates the expression of DBCCR1 via DNA hypermethylation. **a**, **b** The mRNA expression level of DBCCR1 and DBCCR1-003 in Lenti-DBCCR1-003 cells(L-D3) and T24 cells treated by DAC(DAC) compared to the mock and Lenti-vector cells (L-C) were tested by real-time PCR. **c** DBCCR1 protein expression in Lenti-DBCCR1-003 cells and T24 cells treated by DAC compared to the mock and Lenti-vector cells. The intensity of protein bands are quantified.* 1* MOCK;* 2* L-C;* 3* L-D3;* 4* DAC. **d**, **e** MSP analyses of DBCCR1 gene promoter in BC tumor and the matched adjacent tissues of BC. **f** MSP analyses of DBCCR1 gene promoter in Lenti-DBCCR1-003 cells and T24 cells treated by DAC compared to the mock and Lenti-vector cells. Results represent the mean + SD from three independent experiments.* Asterisk* indicate significant difference at p < 0.05

Since the DBCCR1-003 originated from the transcript of DBCCR1, both of them were down-regulated in BC cells and tissues, suggesting that the expression of DBCCR1 may be regulated by DBCCR1-003. To prove that, we knock-in DBCCR1-003 in T24 cells, generating stable cell line Lenti-DBCCR1-003 cells, and tested the expression of DBCCR1-003 and DBCCR1 in Lenti-DBCCR1-003 cells and control counterpart by SYBR real-time PCR. The results indicated that the expression levels of DBCCR1-003 and DBCCR1 in Lenti-DBCCR1-003 cells were higher than those in the mock and Lenti-vector cells (Fig. 2a, b). Further study of Western blot analysis showed that the results were in line with Real time-PCR (Fig. 2c), indicating the expression of DBCCR1 may be regulated by DBCCR1-003. Then, as decrease or lost expression of methylated gene often caused by DNA hypermethylation occurred in carcinogenesis [31], we further hypothesize that DBCCR1-003 regulate the expression of DBCCR1 via DNA methylation. Previously, we found that the promoter region of DBCCR1 was hypermethylated in T24 cells, but unmethylated in SV-HUC-1 cells by MSP. Consistent with the results in cells, 18 out of 24 (75.0 %) samples were methylated in BC tissues, while only 5 out of 24 (20.8 %) samples were methylated in the matched adjacent tissues of BC, suggesting that the methylation status of DBCCR1 in BC was statistical significant higher than controls (Fig. 2d, e; Additional file 1: Fig. S2). Furthermore, by testing the CpG island promoter methylation status of DBCCR1 in Lenti-DBCCR1-003 cells and control counterpart, we found the methylation level of DBCCR1 in Lenti-DBCCR1-003 cells was decreased. Additionally, conducting demethylation experiments on T24 cells, we found the methylation level of DBCCR1 was also decreased but the expression of DBCCR1 was obviously up-regulated compared to the mock control after DAC treatment (Fig. 2b, c, f). Taken together, these results suggest that DBCCR1-003 regulates the expression of DBCCR1 via DNA hypermethylation.

### DNMT1 up-regulation is responsible for the methylation of DBCCR1

**Fig. 3:**
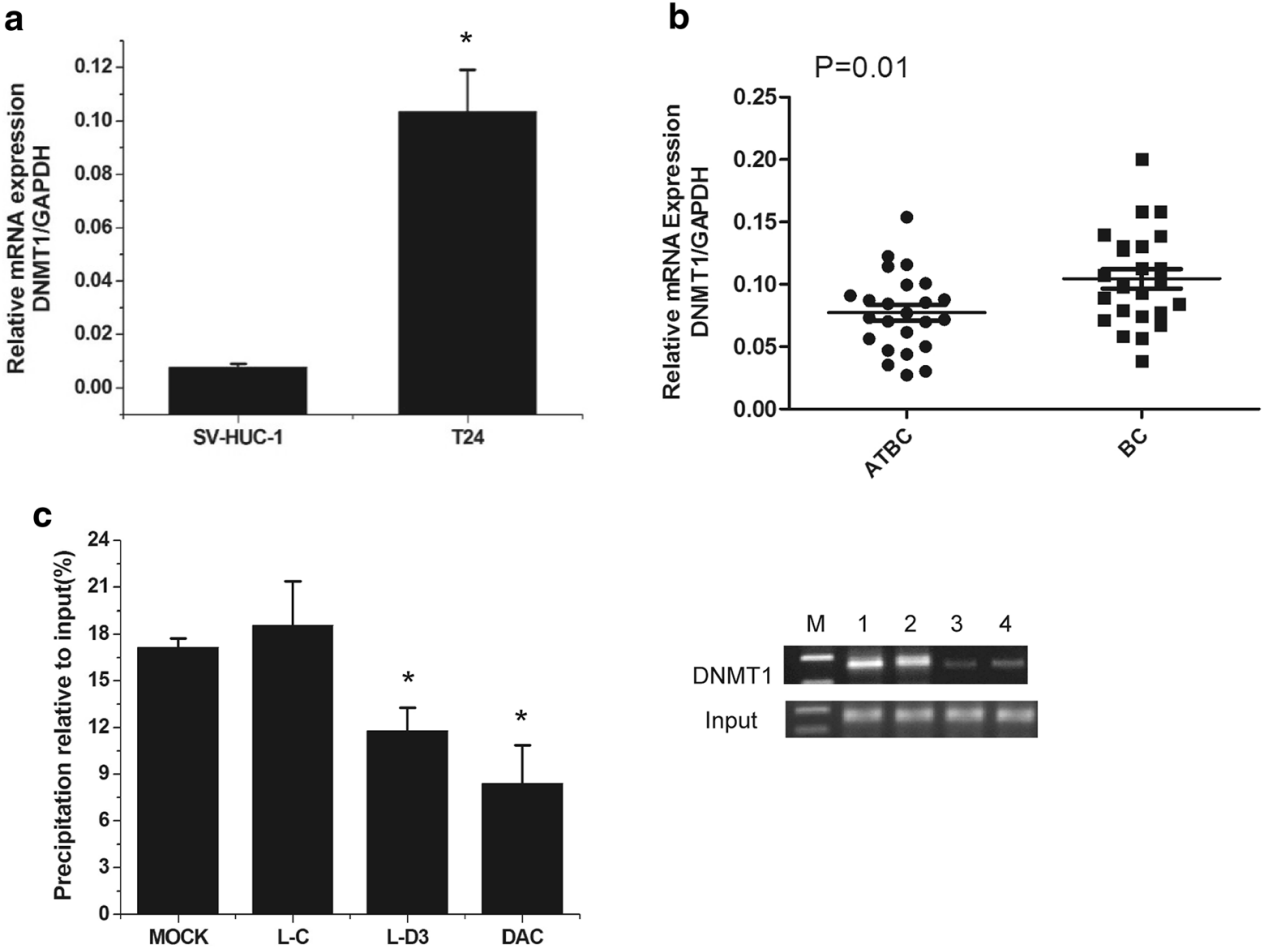
DNMT1 up-regulation is responsible for the methylation of DBCCR1. **a** Relative expression of DNMT1 in T24 cells compared to SV-HUC-1 cells was measured by real-time PCR. **b** Relative expression levels of DNMT1 in 24 pairs of bladder cancer tumors (BC) and the matched adjacent tissues of bladder cancer (ATBT) were determined by real-time PCR. **c** The CHIP analysis of the binding of DNMT1 in DBCCR1 CpG island promoter. Quantitative-CHIP analysis was conducted to measure the binding of DNMT1 in DBCCR1 CpG island promoter with specific antibodies of DNMT1 with normalization by total input DNA.* M* mark;* 1* MOCK; * 2* L-C;* 3* L-D3; *4* DAC. Results represent the mean + SD from three independent experiments. *Asterisk* indicate significant difference at p < 0.05

Previous studies have demonstrated that DNMT1 overexpression may play a key role in the hypermethylation of gene promoter and is related to the malignant potential and poor prognosis of human carcinomatosis [32, 33]. In our study, we found that the expression level of DNMT1 was significant higher in cells and BC tissues (Fig. 3a, b). Since we have found that the methylation level of DBCCR1 was decreased and the expression of DBCCR1 was increased in Lenti-DBCCR1-003 cells and T24 cells treated with DAC, further studies were conducted to determine if DNMT1 was related to the hypermethylation of DBCCR1. The ChIP assay showed that the binding of DNMT1 in DBCCR1 CpG island promoter were obviously decreased in Lenti-DBCCR1-003 cells and T24 cells treated with DAC (Fig. 3c). These results indicated that DNMT1 up-regulation is responsible for the CpG island promoter hypermethylation of DBCCR1.

### DBCCR1-003 may bind to DNMT1 and prevent DNMT1-mediated the methylation of DBCCR1

**Fig. 4:**
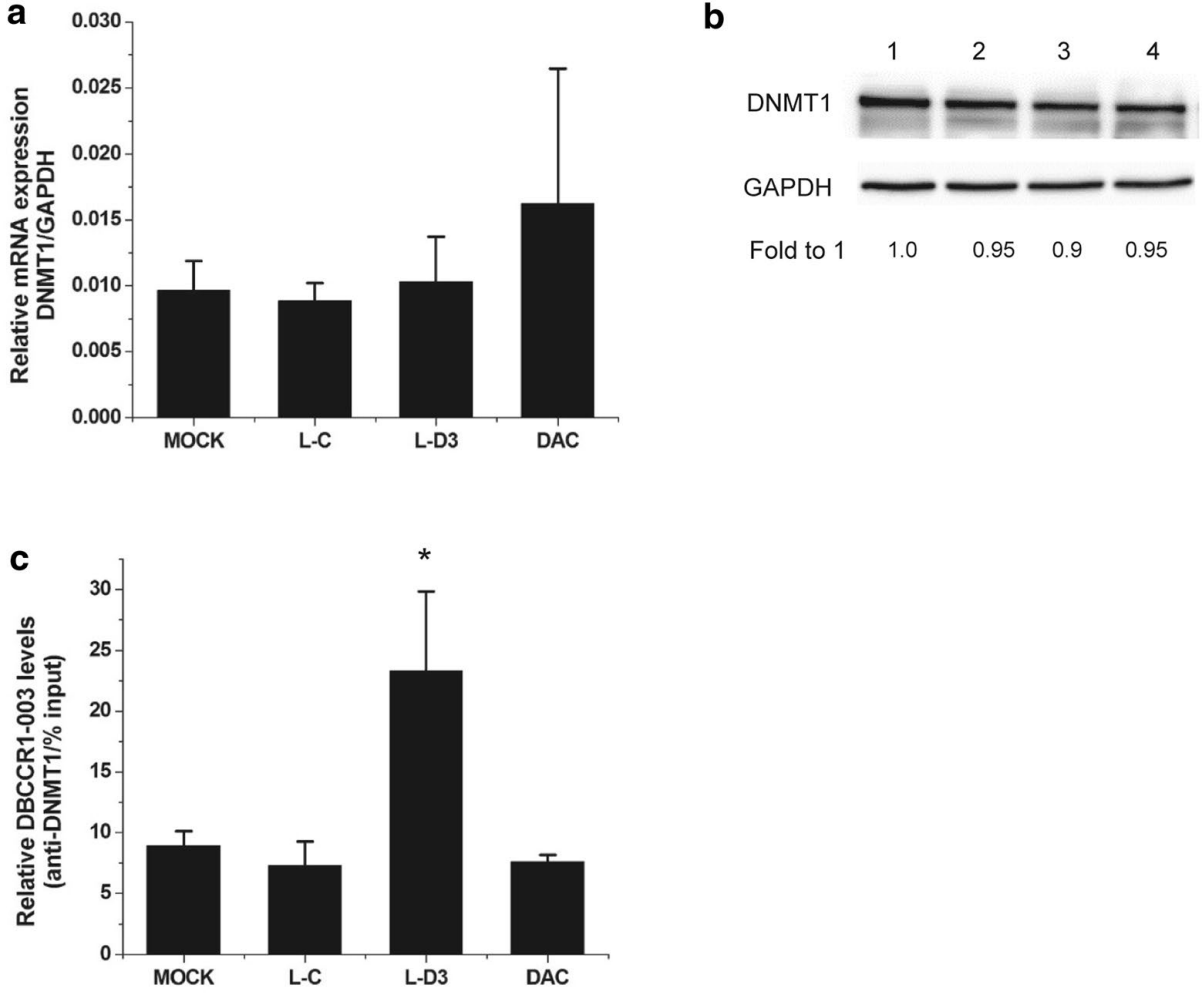
DBCCR1-003 may bind to DNMT1 and prevent DNMT1-mediated the methylation of DBCCR1. **a**, **b** The mRNA and protein expression level of DNMT1 in Lenti-DBCCR1-003 cells (L-D3) and T24 cells treated by DAC (DAC) compared to the mock and Lenti-vector cells (L-C) were tested by real-time PCR and Western Blot, respectively. **c** RT-PCR analysis of DBCCR1-003 in RIP with anti-DNMT1. The enrichment of RNA was measured relative to the input levels using the 2^−△CT^ method. Relative RNA binding levels of DBCCR1-003 and DNMT1 in Lenti-DBCCR1-003 cells and T24 cells treated by DAC compared to the mock and Lenti-vector cells. Results represent the mean + SD from three independent experiments. *Asterisk* indicate significant difference at p < 0.05

Contrary to the relationship between the expression of DBCCR1-003 and the Clinicopathologic features of BC patients, up-regulation level of DNMT1 was found to be significantly correlated with the BC grade and stage, but not patient gender and age (Table 1). However, Spearman’s rank correlation analysis indicated that there was no significant negative correlations existed between the expression levels of DBCCR1-003 and DNMT1 in BC specimens: correlation of DNMT1 with DBCCR1-003 is −0.078 (p = 0.717). What’s more, to test the mRNA and protein levels of DNMT1 in Lenti-DBCCR1-003 cells and T24 cells treated with DAC, Our results indicated that DNMT1 expression did not obviously change in Lenti-DBCCR1-003 cells when compared with control counterpart (Fig. 4a, b). To determine if DBCCR1-003 physically associates with DNMT1 in T24 cells, RIP assay was conducted with specific anti-DNMT1 antibody. The binding of ecCEBPA and DNMT1 which had been identified by studies was used as a positive control to confirm the success of RIP assay. The results indicated that ecCEBPA was enriched in the group of DNMT1 compared to the group of IgG (Additional file 1: Fig. S3). Similarly, DBCCR1-003 was detected in the group of DNMT1 and the enrichment level was obviously increased in Lenti-DBCCR1-003 cells, indicating that DBCCR1-003 may physically associates with DNMT1 in T24 cells (Fig. 4c). Combining with the results that both of the methylation of DBCCR1 and the binding of DNMT1 in DBCCR1 CpG island promoter were decreased in Lenti-DBCCR1-003 cells and T24 cells treated with DAC, suggesting that DBCCR1-003 may bind to DNMT1 and prevent DNMT1-mediated the methylation of DBCCR1 without affecting the expression of DNMT1.

### Effects of DBCCR1-003 on cell growth

**Fig. 5:**
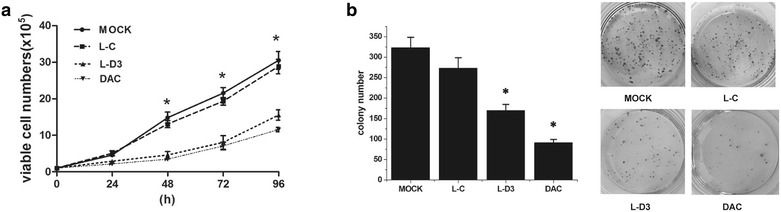
DBCCR1-003 inhibits the growth of T24 cells. **a** Viable cell number was counted each day for 96 h in Lenti-DBCCR1-003 cells (L-D3) and T24 cells treated by DAC compared to the mock and Lenti-vector cells (L-C). **b** The colony numbers was counted after 7 days in Lenti-DBCCR1-003 cells and T24 cells treated by DAC compared to the mock and Lenti-vector cells. The data represent the means ± standard deviations (SDs) from three independent experiments. The *error bars* denote the SDs. *p < 0.05

In order to clarify whether the expression of DBCCR1-003 play a significant role in malignant phenotypes, we performed cell proliferation and clone formation assay to determine the effects of DBCCR1-003 ectopic expression on cell growth in Lenti-DBCCR1-003 cells and control counterpart. The results revealed that the growth of Lenti-DBCCR1-003 cells was obvious reduced compared with that of the Lenti-vector cells. The frequency of colony formation of the Lenti-DBCCR1-003 cells was significantly lower than that of Lenti-vector cells. Further studies in T24 cells treated by DAC and control counterpart also revealed that cells treated by DAC had significantly decreased cell proliferation and a fewer number of colonies formed compared with control (Fig. 5a, b). These results suggested that up-regulation of DBCCR1-003 can inhibit cell growth of T24 cells.

### Effects of DBCCR1-003 on apoptosis and cell cycle distribution

**Fig. 6:**
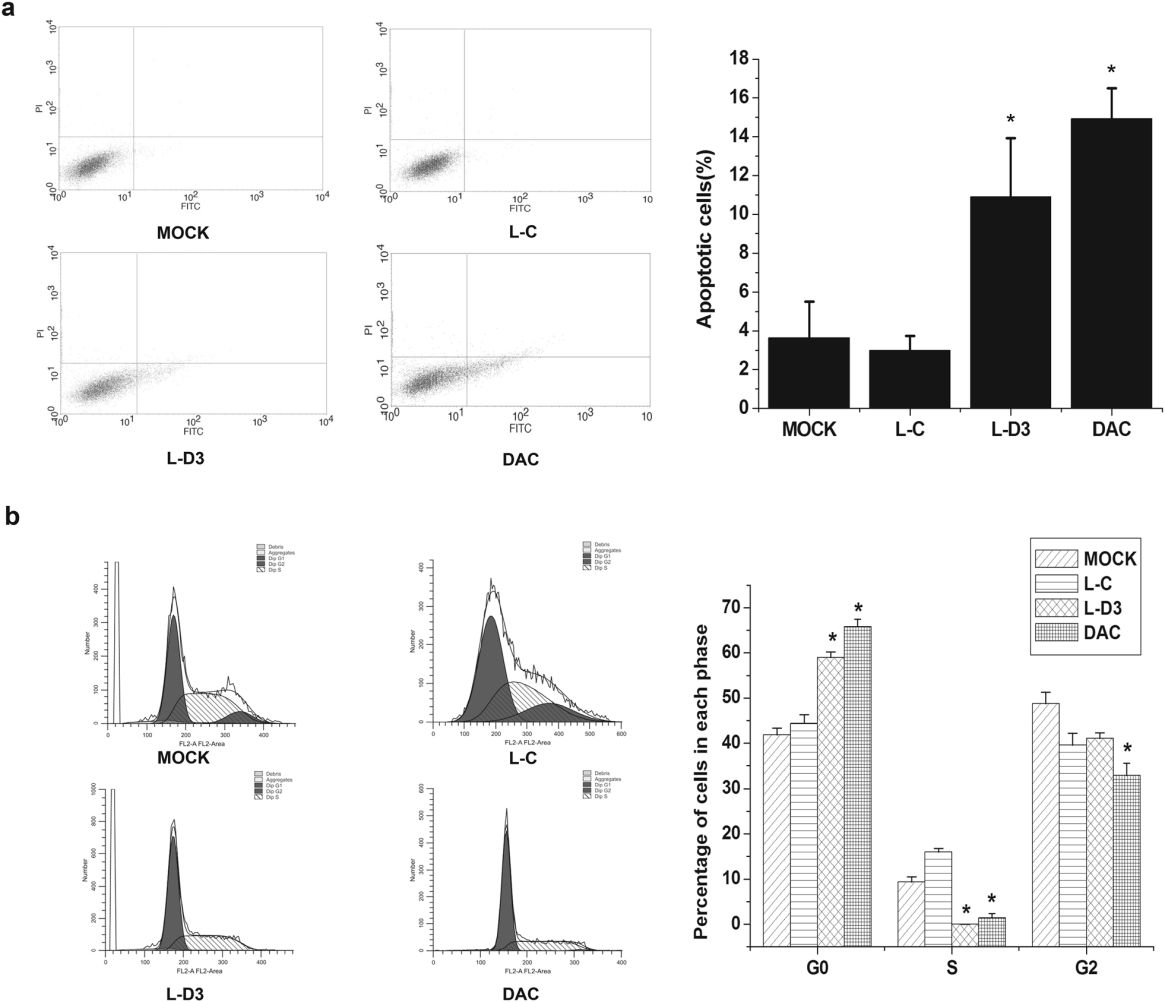
DBCCR1-003 regulated cell cycle progression and apoptosis in T24 cells. **a** The *bar chart* represents the percentage of cells in G0/G1, S, or G2/M phase, as indicated. **b** The percentage of apoptotic cells was determined by flow cytometric analysis. The data represent the mean ± SD from three independent experiments. *p < 0.05

The high expression of DBCCR1-003 can inhibit cell growth in T24 cells, which made us wonder if DBCCR1-003 can trigger apoptosis in T24 cells. To verify this, the effect of DBCCR1-003 on the apoptosis of Lenti-DBCCR1-003 cells, T24 cells treated by DAC and Lenti-vector cells were detected by flow cytometry. The results indicated that the apoptosis rate of Lenti-DBCCR1-003 cells was significantly increased in comparison with that of the Lenti-vector cells. In accordance with these findings, we also found that the percentages of apoptotic cells in T24 cells treated by DAC were as significantly increased compared to the Lenti-vector cells. Taken together, these results suggest that the up-regulation of DBCCR1-003 could induce apoptosis in T24 cells (Fig. 6a).

The above findings suggested that DBCCR1-003 plays an important role in inhibiting cell growth in T24 cells. In order to indentify the possible inhibitory effect mediated by DBCCR1-003, the percentage of Lenti-DBCCR1-003 cells, T24 cells treated by DAC and Lenti-vector cells in different cell cycle phases were determined by the flow cytometry method. The data showed that Lenti-DBCCR1-003 cells and T24 cells treated by DAC significantly decreased the percentage of cells in S phase and increased that in G0/G1 phase compared to Lenti-vector cells (Fig. 6b), indicating that up-regulation of DBCCR1-003 may arrested cell cycle in T24 cells.

## Discussion

Nowadays, there have been raising interests in the role of lncRNAs in human diseases, especially when it involves in the epigenetic modifications. An accumulating number of studies identified that the epigenetic dysregulation of lncRNAs expression in cancer play a key role [7, 17]. In this study, our results indicate that down-regulation of DBCCR1-003 in BC is responsible for the down-regulation of DBCCR1 via DNMT1, and overexpression of DBCCR1-003 can inhibit cell growth by inducing apoptosis and arresting the cell cycle in phase in T24 cells, revealing a new lncRNA DBCCR1-003 which can affect the tumorigenesis and development of BC by mediating tumor suppress gene DBCCR1 via DNMT1.

LncRNAs belong to a versatile group of RNA transcripts without protein-coding potential that function via diverse mechanisms and act as regulator in important biological processes [34]. Taking into account the widespread function that lncRNAs act in cellular networks, there is no surprising that lncRNAs have been involved in human diseases, including the cancer [35]. Studies have indicated that various lncRNAs are related to cellular transformation, having the potential to be tumor suppressors or oncogenes, and leading to tumorigenesis [36]. In gastric cancer, a newly identified lncRNA, CARLo-5, was found to be up-regulated and its knock-down significantly inhibited the cell proliferation [37]. Maternally Expressed Gene 3(MEG3), an imprinted gene that encodes a lncRNA, lost its expression and was negatively associated with tumorigenesis in BC [38]. We found the low expression of DBCCR1-003 in T24 cells and BC tissues, and the cell growth of T24 cells was inhibited by increasing the expression level of DBCCR1-003, indicating that DBCCR1-003 plays a key role in inhibiting BC growth. To explore the possible mechanism responsible for the growth inhibition effect of DBCCR1-003, we performed flow cytometry assay, and found that knock-in DBCCR1-003 induced G0/G1 cell-cycle arrest and cell apoptosis in T24 cells, indicating that BC cell growth mediated by DBCCR1-003 may be related to the regulation of cell cycle and apoptosis. Similar to our results, Shi et al. reported that overexpression of lncRNA, BRAF activated non-coding RNA(BANCR), would suppress colorectal cancer cell growth in vitro and in vivo which was related to induction of G0/G1 cell cycle arrest and apoptosis by regulating p21 [39]. Ma et al. revealed that lncRNA-LET overexpression conferred a inhibition to the cell growth of gallbladder cancer cells through promotion of cell cycle arrest at G0/G1 phase and to the induction of apoptosis under hypoxic conditions [40]. Collectively, our results confirm the tumor-suppressive activity of DBCCR1-003 and suggest that overexpression of DBCCR1-003 inhibits BC growth through the inducing G0/G1 arrest and apoptosis.

In gallbladder cancer, the down-regulation of lncRNA-LET was observed to be associated with poor prognosis, higher tumor status, nodal status, and clinical stage [41]. The similar result was also found in colorectal cancer that lower expression of lncRNA BANCR was related to increased tumor sizes [41]. To DNMT1, DNA hypermethylation on CpG islands is related to the overexpression of DNMT1 in multistage of BC [42]. Consistent with these studies, our results demonstrated that there is the down-regulation of DBCCR1-003 and up-regulation of DNMT1 is related to BC grade and stage.

It has been identified that the dysregulation of lncRNAs is associated with cancer epigenetics [42]. Besides the known and possible epigenetic mechanisms that the lncRNAs involved in cancer can act on tumor suppressor genes, they may associate with a DNA methyltransferase and recruit it to the promoter region of a tumor suppressor gene, leading to the transcriptional silencing of the latter [43]. For example, Li et al. revealed that a lncRNA named AS1DHRS4 (antisense 1 dehydrogenase/reductase SDR family member 4), transcribed from the locus of the DHRS4 gene known to be involved in cancer, modulated the expression of DHRS4 by epigenetic regulation at the DHRS4L2 promoter region [44]. Based on these facts, we hypothesizes that DBCCR1-003 may act as a tumor suppressor gene through regulating the expression of DBCCR1 via DNA methylation. To prove that, we first determined the expression and methylation status of DBCCR1 and found that, like the DBCCR1-003, it was down-regulated and hypermethylation in T24 cells and BC tissues. Then, we knock-in DBCCR1-003 in T24 cells and found that the expression of DBCCR1 was also increased whereas the methylation level of DBCCR1 was decreased. Moreover, treating with DAC in T24 cells, DBCCR1 was up-regulated while the methylation level of DBCCR1’s CpG island was decreased. Meanwhile, similar to the effect of knock-in DBCCR1-003, the cells growth was inhibited and G0/G1 cell-cycle arrest and cell apoptosis was induced in T24 cells treated with DAC. Taken together, these results suggest that DBCCR1-003 may act as a tumor suppressor gene through regulating the expression of DBCCR1 via DNA methylation.

Epigenetic dysregulation of cellular genes is an important feature in the development of human malignancy. More and more evidence has suggested that DNA methylation is among the key players in the human urinary system tumors. The DNA methylation is catalyzed by DNMTs. As one of the three main types of DNMTs (DNMT1,DNMT3A, and DNMT3B)involved in genomic DNA methylation, DNMT1 displays a obvious favour for hemimethylated over unmethylated DNA and maintains DNA methylation [45]. Deepika Dhawan and his coworkers have found that DNMT1 has excellent potential as a target for invasive urothelial carcinoma therapy in human being [46]. The high expression of DNMT1 was demonstrated to play a key role in cell transformation in vitro, suggesting that the abnormal expression of DNMT1 may have influence to the development of human cancer [47]. In our study, we found that the upregulation of DNMT1 expression in T24 cells and BC tissues was related to DBCCR1 low expression with hypermethylation of DBCCR1 promoter. The upregulation of DBCCR1 expression is associated with a decrease of DNA methylation of DBCCR1. Intriguingly, the ChIP assay also identified that the binding of DNMT1 in DBCCR1 CpG island promoter were obviously decreased. These results suggested that DNMT1 up-regulation is responsible for the hypermethylation of DBCCR1. In addition, Wang et al. identified that a novel lncRNA Dum, which modulated DNA methylation by recruiting Dnmts to specific promoter regions, silencing its neighboring gene Dppa2 [48]. Chalei et al. reported that multiple genes could be modulated by a lncRNA Dum to stay away from their site of synthesis through binding to DNMT1 and changing DNA methylation status [49]. Similar to these studies, we found the expression of DNMT1 did not change when DBCCR1-003 was overexpressed. Performing the RIP assay to determine if DBCCR1-003 physically associates with DNMT1 in T24 cells, the results indicated that DBCCR1-003 may bind to DNMT1 in T24 cells. Collectively, the above studies and our results suggest that the expression of DBCCR1 may be regulated by DBCCR1-003 via binding to DNMT1 without affecting the expression of DNMT1 and preventing DNMT1-mediated the methylation of DBCCR1 in BC.

## Conclusions

This study provides the first evidence that overexpression of lncRNA DBCCR1-003 inhibits BC growth through the inducing G0/G1 arrest and apoptosis, and the expression of DBCCR1 may be regulated by DBCCR1-003 via binding to DNMT1 and preventing DNMT1-mediated the methylation of DBCCR1. Tumor suppressor lncRNA DBCCR1-003 may serve as a novel biomarker and therapeutic target for BC in future cancer clinic.

## Additional file

**Additional file 1: Table S1.** Primers used in this study. **Fig. S1**. Schematic view of lncRNAs RUNX3-003 and RASSF1A-011. The region in black indicates the exon and in red indicate the specific sequences of lncRNAs RUNX3-003 and RASSF1A-011. **Fig. S2**. The methylation status of DBCCR1 in tissues. (A, B) MSP analyses of DBCCR1 gene promoter in BC tumor and the matched adjacent tissues of BC. **Fig. S3.** RT-PCR analysis of ecCEBPA in RIP with anti-DNMT1. The enrichment of RNA was measured relative to the input levels using the 2^△CT^ method. Numbers are mean±s.d.(n=3). Relative RNA levels of ecCEBPA in DNMT1 relative to IgG immunoprecipitates.

## Abbreviations

lncRNA: long non coding RNA
BC: bladder cancer
NMIBC: nonmuscle-invasive bladder cancer
MIBC: muscle-invasive bladder cancer
ATBC: adjacent tissues of bladder cancer
DNMT1: DNA methyltransferase 1
DAC: 5-aza-2-deoxycytidine
MSP: methylation-specific PCR
RIP: RNA immunoprecipitation
ChIP: chromatin immunoprecipitation

## References

[1] Kaufman DS, Shipley WU, Feldman AS (2009). Bladder cancer. Lancet.

[2] Prensner JR, Chinnaiyan AM (2011). The emergence of lncRNAs in cancer biology. Cancer Discov.

[3] Mercer TR, Dinger ME, Mattick JS (2009). Long non-coding RNAs: insights into functions. Nat Rev Genet.

[4] Ponting CP, Oliver PL, Reik W (2009). Evolution and functions of long noncoding RNAs. Cell.

[5] Martens-Uzunova ES, Bottcher R, Croce CM, Jenster G, Visakorpi T, Calin GA (2014). Evolution and functions of long noncoding RNAs. Eur Urol.

[6] Guil S, Esteller M (2012). Cis-acting noncoding RNAs: friends and foes. Nat Struct Mol Biol.

[7] Lee JT (2012). Epigenetic regulation by long noncoding RNAs. Science.

[8] Sun M, Jin FY, Xia R, Kong R, Li JH, Xu TP, Liu YW, Zhang EB, Liu XH, De W (2014). Decreased expression of long noncoding RNA GAS5 indicates a poor prognosis and promotes cell proliferation in gastric cancer. BMC Cancer.

[9] Takahashi K, Yan IK, Wood J, Haga H, Patel T (2014). Involvement of extracellular vesicle long noncoding RNA (linc-VLDLR) in tumor cell responses to chemotherapy. Mol Cancer Res.

[10] Ariel I, Sughayer M, Fellig Y, Pizov G, Ayesh S, Podeh D, Libdeh BA, Levy C, Birman T, Tykocinski ML (2000). The imprinted H19 gene is a marker of early recurrence in human bladder carcinoma. Mol Pathol.

[11] Han Y, Liu Y, Gui Y, Cai Z (2013). Long intergenic non-coding RNA TUG1 is overexpressed in urothelial carcinoma of the bladder. J Surg Oncol.

[12] Xue Y, Ma G, Zhang Z, Hua Q, Chu H, Tong N, Yuan L, Qin C, Yin C, Zhang Z (2015). A novel antisense long noncoding RNA regulates the expression of MDC1 in bladder cancer. Oncotarget.

[13] He W, Cai Q, Sun F, Zhong G, Wang P, Liu H, Luo J, Yu H, Huang J, Lin T (2013). linc-UBC1 physically associates with polycomb repressive complex 2 (PRC2) and acts as a negative prognostic factor for lymph node metastasis and survival in bladder cancer. Biochim Biophys Acta.

[14] Cui Z, Ren S, Lu J, Wang F, Xu W, Sun Y, Wei M, Chen J, Gao X, Xu C (2013). The prostate cancer-up-regulated long noncoding RNA PlncRNA-1 modulates apoptosis and proliferation through reciprocal regulation of androgen receptor. Urol Oncol.

[15] Pandey RR, Mondal T, Mohammad F, Enroth S, Redrup L, Komorowski J, Nagano T, Mancini-Dinardo D, Kanduri C (2008). Kcnq1ot1 antisense noncoding RNA mediates lineage-specific transcriptional silencing through chromatin-level regulation. Mol Cell.

[16] Lee JT (2012). Epigenetic regulation by long noncoding RNAs. Science.

[17] Schaukowitch K, Kim TK (2014). Emerging epigenetic mechanisms of long non-coding RNAs. Neuroscience.

[18] Davis CD, Uthus EO (2004). DNA methylation, cancer susceptibility, and nutrient interactions. Exp Biol Med (Maywood).

[19] Liu L, Wylie RC, Andrews LG, Tollefsbol TO (2003). Aging, cancer and nutrition: the DNA methylation connection. Mech Ageing Dev.

[20] Jones PA (1996). DNA methylation errors and cancer. Cancer Res.

[21] Laird PW, Jaenisch R (1994). DNA methylation and cancer. Hum Mol Genet.

[22] Scarano MI, Strazzullo M, Matarazzo MR, D’Esposito M (2005). DNA methylation 40 years later: its role in human health and disease. J Cell Physiol.

[23] Yao J, Zhou B, Zhang J, Geng P, Liu K, Zhu Y, Zhu W (2014). A new tumor suppressor LncRNA ADAMTS9-AS2 is regulated by DNMT1 and inhibits migration of glioma cells. Tumour Biol.

[24] Di Ruscio A, Ebralidze AK, Benoukraf T, Amabile G, Goff LA, Terragni J, Figueroa ME, De Figueiredo PL, Alberich-Jorda M, Zhang P (2013). DNMT1-interacting RNAs block gene-specific DNA methylation. Nature.

[25] Merry CR, Forrest ME, Sabers JN, Beard L, Gao XH, Hatzoglou M, Jackson MW, Wang Z, Markowitz SD, Khalil AM (2015). DNMT1-associated long non-coding RNAs regulate global gene expression and DNA methylation in colon cancer. Hum Mol Genet.

[26] Habuchi T, Luscombe M, Elder PA, Knowles MA (1998). Structure and methylation-based silencing of a gene (DBCCR1) within a candidate bladder cancer tumor suppressor region at 9q32-q33. Genomics.

[27] Habuchi T, Takahashi T, Kakinuma H, Wang L, Tsuchiya N, Satoh S, Akao T, Sato K, Ogawa O, Knowles MA (2001). Hypermethylation at 9q32-33 tumour suppressor region is age-related in normal urothelium and an early and frequent alteration in bladder cancer. Oncogene.

[28] Herman JG, Graff JR, Myohanen S, Nelkin BD, Baylin SB (1996). Methylation-specific PCR: a novel PCR assay for methylation status of CpG islands. Proc Natl Acad Sci USA.

[29] Lee MG, Kim HY, Byun DS, Lee SJ, Lee CH, Kim JI, Chang SG, Chi SG (2001). Frequent epigenetic inactivation of RASSF1A in human bladder carcinoma. Cancer Res.

[30] Kim WJ, Kim EJ, Jeong P, Quan C, Kim J, Li QL, Yang JO, Ito Y, Bae SC (2005). RUNX3 inactivation by point mutations and aberrant DNA methylation in bladder tumors. Cancer Res.

[31] Wilson AS, Power BE, Molloy PL (2007). DNA hypomethylation and human diseases. Biochim Biophys Acta.

[32] Bernardino J, Roux C, Almeida A, Vogt N, Gibaud A, Gerbault-Seureau M, Magdelenat H, Bourgeois CA, Malfoy B, Dutrillaux B (1997). DNA hypomethylation in breast cancer: an independent parameter of tumor progression?. Cancer Genet Cytogenet.

[33] Soares J, Pinto AE, Cunha CV, Andre S, Barao I, Sousa JM, Cravo M (1999). Global DNA hypomethylation in breast carcinoma: correlation with prognostic factors and tumor progression. Cancer.

[34] Wang X, Song X, Glass CK, Rosenfeld MG (2011). The long arm of long noncoding RNAs: roles as sensors regulating gene transcriptional programs. Cold Spring Harb Perspect Biol.

[35] Esteller M (2011). Non-coding RNAs in human disease. Nat Rev Genet.

[36] Gutschner T, Diederichs S (2012). The hallmarks of cancer: a long non-coding RNA point of view. RNA Biol.

[37] Zhang Y, Ma M, Liu W, Ding W, Yu H (2014). Enhanced expression of long noncoding RNA CARLo-5 is associated with the development of gastric cancer. Int J Clin Exp Pathol.

[38] Ying L, Huang Y, Chen H, Wang Y, Xia L, Chen Y, Liu Y, Qiu F (2013). Downregulated MEG3 activates autophagy and increases cell proliferation in bladder cancer. Mol BioSyst.

[39] Shi Y, Liu Y, Wang J, Jie D, Yun T, Li W, Yan L, Wang K, Feng J (2015). Downregulated long noncoding RNA BANCR promotes the proliferation of colorectal cancer cells via downregulation of p21 Expression. PLoS One.

[40] Ma MZ, Kong X, Weng MZ, Zhang MD, Qin YY, Gong W, Zhang WJ, Quan ZW (2014). Long non-coding RNA-LET is a positive prognostic factor and exhibits tumor-suppressive activity in gallbladder cancer. Mol Carcinog.

[41] Nakagawa T, Kanai Y, Ushijima S, Kitamura T, Kakizoe T, Hirohashi S (2005). DNA hypermethylation on multiple CpG islands associated with increased DNA methyltransferase DNMT1 protein expression during multistage urothelial carcinogenesis. J Urol.

[42] Morris KV (2009). Long antisense non-coding RNAs function to direct epigenetic complexes that regulate transcription in human cells. Epigenetics.

[43] Beckedorff FC, Amaral MS, Deocesano-Pereira C, Verjovski-Almeida S (2013). Deocesano-Pereira C, Verjovski-Almeida S. Long non-coding RNAs and their implications in cancer epigenetics. Biosci Rep.

[44] Li Q, Su Z, Xu X, Liu G, Song X, Wang R, Sui X, Liu T, Chang X, Huang D (2012). AS1DHRS4, a head-to-head natural antisense transcript, silences the DHRS4 gene cluster in cis and trans. Proc Natl Acad Sci USA.

[45] Bestor TH (2000). The DNA methyltransferases of mammals. Hum Mol Genet.

[46] Dhawan D, Ramos-Vara JA, Hahn NM, Waddell J, Olbricht GR, Zheng R, Stewart JC, Knapp DW (2013). DNMT1: an emerging target in the treatment of invasive urinary bladder cancer. Urol Oncol.

[47] Szyf M, Detich N (2001). Regulation of the DNA methylation machinery and its role in cellular transformation. Prog Nucleic Acid Res Mol Biol.

[48] Wang L, Zhao Y, Bao X, Zhu X, Kwok YK, Sun K, Chen X, Huang Y, Jauch R, Esteban MA (2015). LncRNA Dum interacts with Dnmts to regulate Dppa2 expression during myogenic differentiation and muscle regeneration. Cell Res.

[49] Chalei V, Sansom SN, Kong L, Lee S, Montiel JF, Vance KW, Ponting CP (2014). The long non-coding RNA Dali is an epigenetic regulator of neural differentiation. Elife.

