# Supplementary material for: Long non-coding RNA DBCCR1-003 regulate the expression of DBCCR1 via DNMT1 in bladder cancer
Source: Cancer Cell Int. 2016 Oct 18;16:81. doi: 10.1186/s12935-016-0356-8 (PMC5069854; doi:10.1186/s12935-016-0356-8)
Supplement: Supplementary file 1 — Additional file 1: Table S1. Primers used in this study. Fig. S1. Schematic view of lncRNAs RUNX3-003 and RASSF1A-011. The region in black indicates the exon and in red indicate the specific sequences of lncRNAs RUNX3-003 and RASSF1A-011. Fig. S2. The methylation status of DBCCR1 in tissues. (A, B) MSP analyses of DBCCR1 gene promoter in BC tumor and the matched adjacent tissues of BC. Fig. S3. RT-PCR analysis of ecCEBPA in RIP with anti-DNMT1. The enrichment of RNA was measured relative to the input levels using the 2△CT method. Numbers are mean±s.d.(n=3). Relative RNA levels of ecCEBPA in DNMT1 relative to IgG immunoprecipitates. [file 12935_2016_356_MOESM1_ESM.docx]

**Table 1.**Primers used in this study

| **Primer Name** | **Primer sequence(5’——3’)** | |
| --- | --- | --- |
|  | **F** | **R** |
| **RT-PCR Primers** |  |  |
| DBCCR1 | CAACGCACTGCCCGCAAGCTT | TGTTCCCGCCTATCACGCAGG |
| DBCCR1-003 | CAGAGACAAGGCAAGATTGAAAACA | GGGGACGGAGGAAAAGAGGAT |
| RUNX3 | AGCACCACAAGCCACTTCAG | GGGAAGGAGCGGTCAAACTG |
| RUNX3-003 | GTTGGCTCCTGGTGCTCC | GGGAGGGAGGTGTGAAGC |
| RASSF1A | TCTGGGGCGTCGTGCGCAAA | GAACCTTGATGAAGCCTGTG |
| RASSF1A-011 | TGTTTTGGACCATAAGCATTCAG | TCTCCGATAATGGGGATACCTAG |
| DNMT1 | ACGACCCTGACCTCAAATAT | CCATTAACACCACCTTCAAGA |
| GAPDH | GCACCGTCAAGGCTGAGAAC | TGGTGAAGACGCCAGTGGA |
| **MSP Primers** |  |  |
| DBCCR1(Methylation) | TTGTAAATTGATTTGGCGCGC | TTCCGAACACGACGCGAAA |
| DBCCR1(No Methylation) | TTTATGGTTGTAAATTGATTTGGTGTGT | CAACTCACATTCCAAACACAACACA |
| RUNX3(Methylation) | GAGGGGCGGTCGTACGCGGG | AAAACGACCGACGCGAACGCCTCC |
| RUNX3 (No Methylation) | GAGGGGTGGTTGTATGTGGG | AAAACAACCAACACAAACACCTCC |
| RASSF1A (Methylation) | GGGTTTTGCGAGAGCGCG | GCTAACAAACGCGAACCG |
| RASSF1A (No Methylation) | GGTTTTGTGAGAGTGTGTTTAG | CACTAACAAACACAAACCAAAC |
| **ChIP Primer** |  |  |
| DBCCR1 promoter | CGCACTTGGACTTCCCTCT | AAATGGCAGTGTCAGTTGTA |
| **RIP Primer** |  |  |
| ecCEBPA | TCATGAGCTCTCCATCCATCCTGA | CTGGCCGAGGGTCCTGCTGGAATC |
| DBCCR1-003 | CAGAGACAAGGCAAGATTGAAAACA | GGGGACGGAGGAAAAGAGGAT |


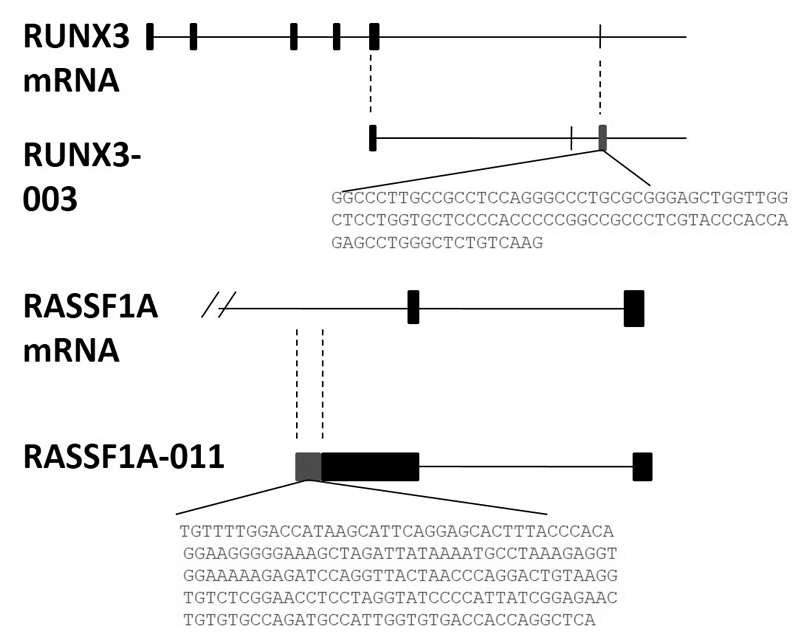


Fig.1-Schematic view of lncRNAs RUNX3-003 and RASSF1A-011. The region in black indicates the exon and in red indicate the specific sequences of lncRNAs RUNX3-003 and RASSF1A-011.


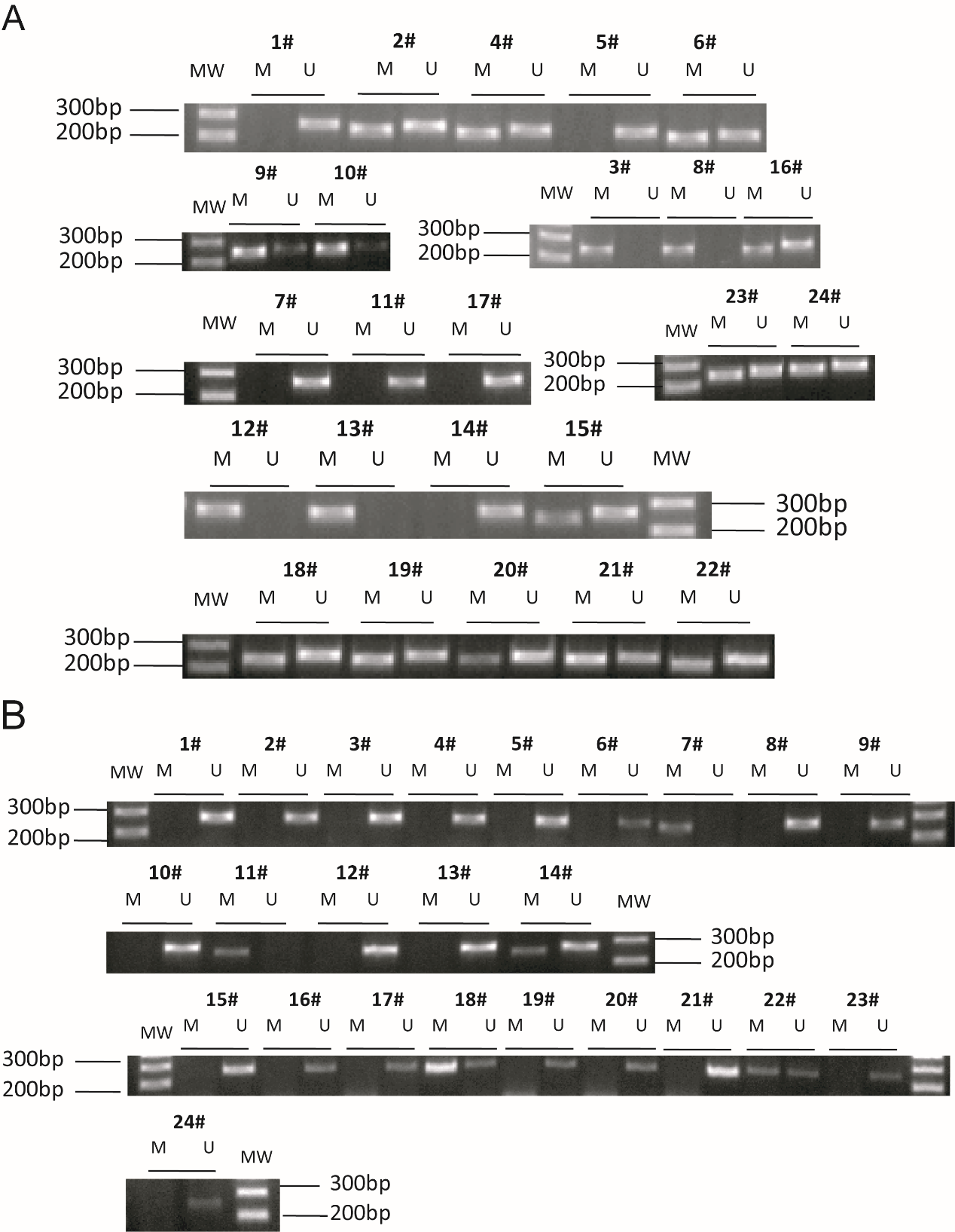


Fig.2-The methylation status of DBCCR1 in tissues. (A, B) MSP analyses of DBCCR1 gene promoter in BC tumor and the matched adjacent tissues of BC.


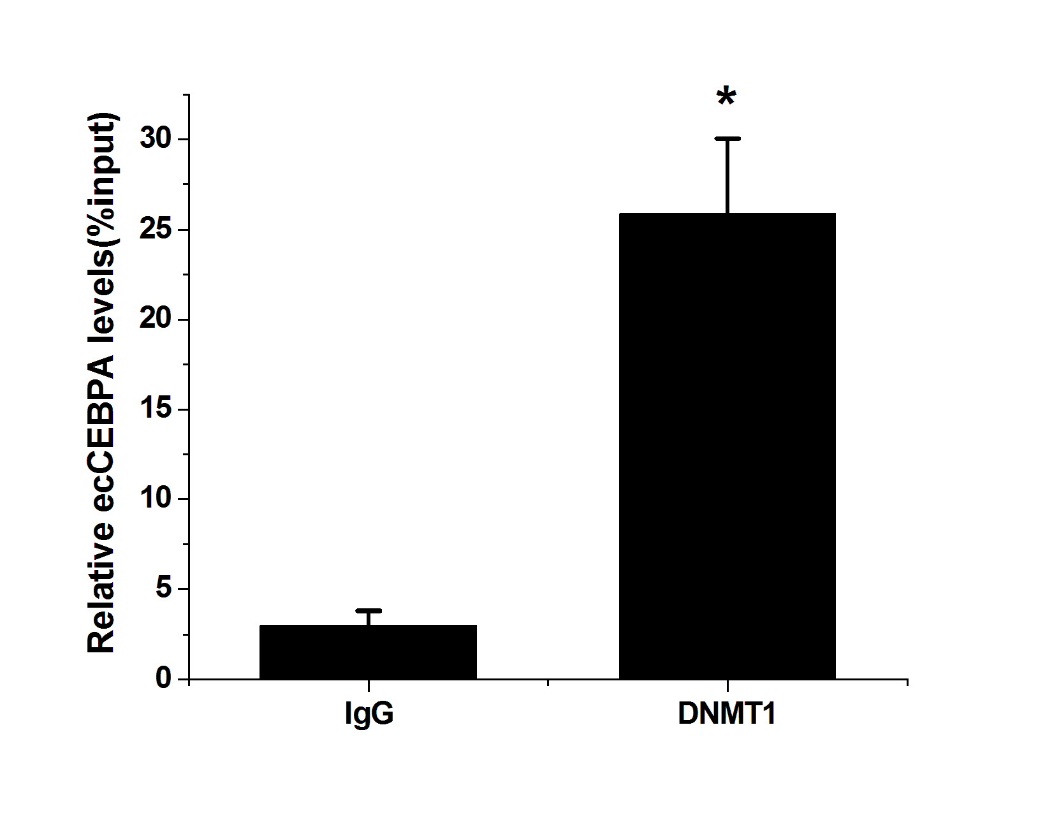


Fig.3-RT-PCR analysis of ecCEBPA in RIP with anti-DNMT1. The enrichment of RNA was measured relative to the input levels using the 2^△CT^ method. Numbers are mean±s.d.(n=3). Relative RNA levels of ecCEBPA in DNMT1 relative to IgG immunoprecipitates.
